# Association between Screen Time and Sociodemographic Factors, Physical Activity, and BMI among Children in Six European Countries (Feel4Diabetes): A Cross-Sectional Study

**DOI:** 10.3390/children11040458

**Published:** 2024-04-11

**Authors:** Sándor Istvánné Radó, Mónika Molnár, Róbert Széll, Gergő József Szőllősi, Viktória Törő, Bashar Shehab, Yannis Manios, Costas Anastasiou, Violeta Iotova, Kaloyan Tsochev, Nevena Chakarova, Natalia Giménez-Legarre, Maria Luisa Miguel Berges, Peter E. H. Schwarz, Imre Rurik, Attila Sárváry

**Affiliations:** 1 Department of Nursing and Midwifery, Faculty of Health Sciences, University of Debrecen, 4400 Nyíregyháza, Hungary; rado.sandorne@etk.unideb.hu (S.I.R.); toro.viktoria@etk.unideb.hu (V.T.); 2 Doctoral School of Health Sciences, University of Debrecen, 4032 Debrecen, Hungary; basharshehab71@gmail.com; 3 Department of Integrative Health Sciences, Faculty of Health Sciences, University of Debrecen, 4400 Nyíregyháza, Hungary; molnar.monika@etk.unideb.hu (M.M.); szell.robert@etk.unideb.hu (R.S.); 4 Coordination Center of Social Sciences, Faculty of Economy, University of Debrecen, 4032 Debrecen, Hungary; szollosi.gergo@etk.unideb.hu; 5 Department of Nutrition and Dietetics, School of Health Science & Education, Harokopio University, 176 76 Athens, Greece; manios@hua.gr (Y.M.); acostas@hua.gr (C.A.); 6 Department of Pediatrics and Medical Genetics, Medical University of Varna, 9002 Varna, Bulgaria; violeta.iotova@mu-varna.bg (V.I.); kalooyan@abv.bg (K.T.); 7 Department of Endocrinology, Medical University of Sofia, 1431 Sofia, Bulgaria; nchakarova@medfac.mu-sofia.bg; 8 GENUD (Growth, Exercise, Nutrition and Development) Research Group, Facultad de Ciencias de la Salud, Universidad de Zaragoza, 50009 Zaragoza, Spain; nglegarre@unizar.es (N.G.-L.); mlmiguel@unizar.es (M.L.M.B.); 9 Instituto Agroalimentario de Aragón (IA2), 50013 Zaragoza, Spain; 10 Instituto de Investigación Sanitaria Aragón (IIS Aragón), 50009 Zaragoza, Spain; 11 Centro de Investigación Biomédica en Red de Fisiopatología de la Obesidad y Nutrición (CIBERObn), Instituto de Salud, Carlos III, 28029 Madrid, Spain; 12 International Diabetes Federation, Medical Faculty Carl Gustav Carus, Technical University of Dresden, 01069 Dresden, Germany; peter.schwarz@uniklinikum-dresden.de; 13 Department of Family Medicine, Semmelweis University, 1085 Budapest, Hungary

**Keywords:** screen time, parental education, body mass index, physical activity, income status

## Abstract

Screen time among children in most European countries is notably high and is influenced by various sociodemographic and other factors. Our study aimed to explore the associations between parents’ sociodemographic characteristics, socioeconomic status, body mass index (BMI), physical activity, risk status for type 2 diabetes, and their children’s BMI, physical activity, and screen time. The data were sourced from the 2016 Feel4Diabetes study, involving 12,280 parents and 12,211 children aged 6–9 years (average age 8.21 years) in a cross-sectional study design. We used a logistic regression model to identify potential factors associated with children’s screen time. The results showed that mothers with tertiary education (OR = 0.64; 95%CI = 0.49–0.82; *p* < 0.001), the middle age group (45–54 years) (OR = 0.81 95%CI = 0.66–0.98; *p* = 0.033), and families with higher incomes (middle–OR = 0.85; 95%CI = 0.75–0.97; *p* = 0.014; high–OR = 0.8; 95%CI = 0.69–0.93; *p* = 0.003) were associated with a decreased chance of children spending more than 2 h/day in front of the screen. In contrast, maternal overweight/obesity (OR = 1.15; 95%CI = 1.03–1.29; *p* = 0.013) and lower physical activity in children were linked to an increased likelihood of more than 2 h of screen time per day. Our findings suggest that targeted interventions should be developed to mitigate excessive screen time, particularly focusing on low-income families and mothers with low educational levels.

## 1. Introduction

Today, the widespread use of electronic media devices has resulted in their use starting at an increasingly young age and becoming more common among children. It is common that children’s exposure to screens is present from a very early stage of their development [1,2,3,4,5,6,7,8,9,10,11,12]. In recent years, in addition to the previously dominant screen-based activity of television, newer devices such as computers, video games, tablets, and smartphones have appeared. Studies have found that excessive screen use by children is associated with a range of adverse consequences [2,4,8,12,13,14,15]. Increased screen use among children has become so significant that addressing the adverse effects on their development is of paramount importance as a public health problem [3,16,17,18]. Excessive screen time is known to have a negative impact on children’s physical [1,4,5,6,8,12,15,16,19], cognitive [1,2,3,8,11,15], and language [1,2,7,9,15] development, as well as contributing to obesity [1,4,5,6,7,8,10,11,12,13,14,15,16,17,18,20,21,22], sleep problems [1,3,4,7,8,13], and mental and psychological disorders [1,3,4,5,6,7,8,9,14,15,16,19,22,23]. International health organizations have set a guideline of 2 h per day [1,3,6,14,18], but this recommended limit is not respected by a high percentage of parents for children under 2–3 years [2,6,15,17], and in school-age children, it is often exceeded by many times the guideline [2,3,6,15,17]. In European countries, 60% of children aged 6–9 years spend more than two hours in front of a screen [24].

In our review of the summary research, we found that the issue is much more complex than it first appears. For example, the need to distinguish between different screens is critical, as their effects can vary [1,15,17]. The majority of previous research has focused on TV viewing. In this context, children’s screen time was strongly influenced by whether they had their own TV set in their room, a situation that has been exacerbated by the proliferation of mobile devices [1,3,5,12,15,17,21,24]. The mechanisms of screen time are influenced by children’s age, media content, pace [1,3,9], family environment [3,7,9,22], and presumably many other factors. Logically, as the time spent in front of the screen increases, children’s physical activity decreases inevitably, leading to a high prevalence of sedentary behavior. However, research has already shown that the negative effects of screen time and sedentary behavior are not compensated by physical activity [1,19]. A link has been found between children’s physical activity and screen time, but screen use is associated with far more health risks than simply being sedentary [4,5,11,12,13,19,20,24,25]. Increased screen use has been associated with an increase in children’s body mass index (BMI), meaning they are more likely to develop high blood pressure, high insulin levels, or high cholesterol levels by adolescence [2,3,12,13,17,20]. Studies have reported a significant dose-response relationship between type 2 diabetes [3,10,12,22], cardiovascular disease [3,4,10,12,13,15,22], and time spent in front of a TV screen both in children and adults.

The family environment, including parental attitude, clearly influences children’s health behavior, affecting their quality of life [3,7,9,22,26]. Previous research on screen use among children under 3 years of age has found no association between the father’s education or mother’s BMI, employment, and screen use of these children. There was no clear association with the mother’s age, education, and household income [2]. An international study of children aged 4–17 years found a significant association between children’s gender (higher screen time for boys), age (different associations in different countries), mother’s education (lower for those with university education), children’s BMI, and screen time over 2 h [16]. Since electronic device use among the young generation rapidly changed in the last few years [27,28], it is important to reveal the factors contributing to excessive usage to develop and implement effective preventive measures. Our study sample contains respondents from developing and developed countries; therefore, it can provide a unique opportunity to examine sociodemographic and health behavior factors presumably associated with screen time.

The aim of our study was to explore the relationship between parents’ (caregivers’) sociodemographic and socioeconomic factors, body mass index, physical activity, risk group for type 2 diabetes, and children’s screen time. We also investigated the impact of children’s gender, BMI, and physical activity on their screen time.

## 2. Materials and Methods

### 2.1. Study Background and Data Collection

The study we describe used cross-sectional data from the EU-funded Feel4Diabetes study, which aimed to develop and implement a school- and community-based intervention to help reduce the risk of type 2 diabetes mellitus (T2DM), with a particular focus on modifiable risk factors [29]. The study has been registered in clinical trials under the study number NCT02393872. The Feel4Diabetes study adhered to the principles outlined in the Declaration of Helsinki and the Council of Europe’s Conventions on Human Rights and Biomedicine. All participating countries obtained the necessary ethical research approval to conduct the study.

The research involved six European countries: Belgium, Finland, Greece, Spain, Bulgaria, and Hungary. The participating countries were further clustered according to their economic status, with high-income countries (Belgium and Finland), countries under austerity (Greece and Spain), and low-income countries (Bulgaria and Hungary) [30]. Data were collected between April and September 2016. Primary schools served as the entry point for the community component, where participants were recruited using a standardized, multi-stage sampling procedure. All areas were included in low-income countries, while in high-income countries or countries under austerity, areas with low socioeconomic status (based on educational disadvantage and unemployment rates) were targeted.

Children in the first three years of primary education and their families were recruited from these schools to take part in the study. Children took home an information letter to their families, briefly informing parents about the purpose of the study. Parents gave their permission to participate in the study by signing a written consent form. The consenting parents were then asked to complete a questionnaire, and the researchers visited the schools concerned to measure the children’s weight and height objectively. Further details of this research and the data collection can be found elsewhere [31]. A detailed description of the study has been previously provided [32].

### 2.2. Measures

The questionnaire developed for the Feel4Diabetes study was completed by the parent/caretaker for themselves and their child. The questionnaire included sociodemographic data, physical activity, dietary, and screen time characteristics. Additionally, parents were asked to complete the Findrisc questionnaire, which consists of eight scored questions and measures the 10-year risk of developing T2DM [33]. Based on the Findrisc results, families were classified as high risk (HR) for developing T2DM if at least one parent had a Findrisc score of a minimum of 9. For the present study, only relevant sociodemographic data (age, gender, education, and family livelihood characteristics) and data on children’s and parents’ screen time and physical activity were used. Only data provided by parents/guardians of primary school-age children aged 6 to 9 years were included in the present study. Participants with incomplete data on outcome variables or no data on screen time were also excluded from the dataset.

#### 2.2.1. Body Mass Index

Parents’ BMI was calculated from self-reported questionnaire results and classified as normal up to 25 kg/m^2^, overweight from 25 to 30 kg/m^2^, and obese above 30 kg/m^2^ according to the WHO (World Health Organization) criteria. The children were measured in schools by the research assistants mentioned earlier. Height was measured with a Seca 2017 stadiometer and weight with a Seca 813 digital scale. The resulting data were used to calculate the Z-score for the under-18 age group, as recommended by the International Obesity Task Force (IOTF) [34]. The Z-score can be derived from the percentiles corresponding to the child’s weight, height, sex, and age. Based on the results obtained, children were classified according to the IOTF cut-off scores: −1 or below (underweight), −1 to +1 (normal), +1 to +2 (overweight), and +2 and above (obese).

#### 2.2.2. Education Level

To determine the educational attainment of parents, we used the ISCED (International Standard Classification of Education) index [35]. Based on the years of schooling completed, if at least one parent had it, we classified respondents into the categories of low (9 years or less), intermediate (10–14 years), and higher (15 years or more) education.

#### 2.2.3. Physical Activity

Information on the physical activity of parents and children was examined through inquiries. Adults were asked, “In the past week, how many times did you engage in at least 30 min of physical activity a day?” Parents were asked, “In the past week, how many times did your child engage in at least 1 h of physical activity per day?” Physical activity is defined as any activity that raises your heart rate or makes you sweat a little, such as walking, cycling, team sports, aerobics, dancing, gardening, or any other similar activity. Responses were recorded separately for weekend and weekday activities. For weekdays, response options ranged from “1”, indicating no activity, to “6”, indicating activity on all five weekdays, while for weekends, options ranged from “1”, indicating no activity, to “3”, indicating activity on both days. The analysis was based on recommendations from the WHO, the Canadian Sedentary Behavior Guidelines [36], and the Canadian 24-Hour Movement Guidelines for Adults [37]. Regarding the time spent with physical activity, we categorized the adults’ and children’s values into two groups according to the guidelines (minimum 150 min per week for adults and 7 h per week for children).

#### 2.2.4. Screen Time

We examined children’s and adults’ screen time (excluding time spent at school or work) on weekdays and weekends. “Screen time” was defined as the following activities: watching TV/DVD, using a PC, mobile phone, or tablet, and playing video games. Response categories ranged from 0 on weekdays to 10 on weekends. The lowest category ranged from “Never” to more than 7 h per day. The categories were analyzed using recommendations from the WHO, the IDEFICS (Identification and Prevention of Dietary- and Lifestyle-Induced Health Effects in Children and Infants) [38] study, and the American Academy of Pediatrics [39]. These organizations recommended a maximum acceptable level of screen time for adults and children of 2 h per day.

#### 2.2.5. Income

A six-point Likert scale based on self-report was used to assess families’ livelihoods, with a range from 1 to 6, where 1 indicated “Very difficult”, and 6 indicated “Very easy”. Based on this scale, the results were processed by classifying the respondents into three categories: “Low”, “Average”, and “High” quality of livelihood families.

### 2.3. Statistical Methods

A Pearson chi-square test was performed on the resulting database to assess unproven differences in categorical characteristics between the participants’ pre-monitor activity matching and non-matching groups. Continuous variables were presented with medians and interquartile ranges. A multivariate logistic regression model was performed to identify factors that might influence screen time in the study group. We analyzed interactions between explanatory variables, identifying six potentially significant pairs. Results are presented as adjusted odds ratios (OR) and *p*-values. Statistical analysis was performed using Stata statistical software (version 13.0, Stata Corp, College Station, TX, USA), and *p* < 0.05 was considered significant.

## 3. Results

### 3.1. The Pattern Shown Using Descriptive Statistics

In the original 2016 survey, 12,280 families completed both parts of the Feel4Diabetes (F4D) questionnaire. The distribution of responding families is as follows: after analyzing the data from the Findrisc questionnaire completed by the families surveyed, 2763 (22%) families were classified as high risk and 9517 (78%) as low or less at risk of T2DM.

The age distribution of mothers was as follows: <45 years, 10,651 (90%); 45–54 years, 1113 (8%); 55–64 years, 18 (1.5%); and over 64 years, 5 (0.5%). The sample of mothers who self-reported as normal weight (<25 kg/m^2^) was 7778 (67%), and overweight or obese (>25 kg/m^2^) was 3870 (33%). In terms of educational attainment, the majority of mothers had tertiary, college, or university education, totaling 6373 (56%).

In terms of fathers’ age, there were 7885 (75%) fathers aged under 45 years, 2066 (20%) aged between 45 and 54 years, 182 (1.8%) aged 55–64 years, and 20 (0.2%) aged over 64 years. Fathers’ self-reported BMI fell within the normal category (<25 kg/m^2^) for 3146 (32%), while 6828 (68%) were classified as overweight or obese (≥25 kg/m^2^). In terms of educational attainment, 46% of fathers had tertiary education.

According to their own assessment, 5703 (50%) families rated their daily living standard as low, 3302 (29%) as medium, while 2501 (21%) considered themselves to have a high or good standard of living. When the physical activity of parents is analyzed, 4098 (45%) of them meet the recommended level of physical activity (150 min per week), while 4958 (55%) consider themselves to have no physical activity.

The average age of the 12,211 children was 8.21 years (Mdn = 8.16 [7.46–8.93]), and the sex ratio was 6180 (51%) girls and 6031 (49%) boys (Table 1). The distribution of children’s body mass index (BMI) Z-score classification was 0.56 (Mdn = 0.47 [−1.98, 0.19–1.29]). Regarding physical activity, 62% of children engage in at least 1 h of physical activity per day, while 38% do not meet the minimum guidelines.

For screen time, which was the main focus of our research, we found that 4283 (47%) of children adhered to the recommended maximum of 2 h per day, while 53% had screen time of more than 2 h per day.

### 3.2. Cross-Table Analysis of Screen Time

Among the six countries we surveyed, 47% of respondents (*n* = 4283) reported adhering to the recommended screen time, while in Belgium (*n* = 771, 57%), Finland (*n* = 734, 57%), and Bulgaria (*n* = 1298, 59%), the respondents spent more than 2 h per day in front of a screen (*p* < 0.001) (Table 2).

Of the children whose families were not at increased risk of T2DM (*n* = 7031), 3358 (48%) had adequate screen time, while 3673 (52%) did not. No significant difference was found between the two groups (*p* = 0.192). No significant association was found between age groups and children’s screen time for either mothers or fathers. Excessive screen time was significantly higher among children of overweight or obese mothers (*n* = 1203, 44%) compared to those with mothers of normal BMI (*n* = 2896, 49%) (*p* < 0.001). No significant association was found between fathers’ BMI and their children’s screen time. The proportion of children with unfavorable screen time was significantly lower among children of mothers and fathers with tertiary education (*p* < 0.001). Considering the livelihood categories of families, we found a significant association with children’s screen time. The proportion of children with excessive screen time was lower in families with high and middle income than those in low-income families (*p* = 0.035).

No significant association was found between the physical activity of adult respondents and the corresponding screen time of children (*p* = 0.996).

We also found no significant association between children’s gender or physical activity and children’s time spent in front of a screen (*p* = 0.484; *p* = 0.881).

### 3.3. Multivariate Model

Compared to Hungarian children, Belgian children were 86% more likely (OR = 1.86, *p* < 0.001), Finnish children 76% more likely (OR = 1.76, *p* < 0.001), and Bulgarian children 91% more likely (OR = 1.91, *p* < 0.001) to have negative screen time outcomes (Table 3).

In relation to parents’ age group and children’s screen time, children whose mothers were in the 45–54 age group were significantly less likely (OR = 0.81, *p* = 0.033) to spend more than 2 h in front of a screen compared to children whose mothers were in the younger age group (<45 years). No significant association was found for fathers. 

Examining the association between parental BMI and children’s screen time, it was found that compared to parents with a normal BMI, the proportion of children with high screen time was significantly higher among mothers with high BMI (OR = 1.15, *p* = 0.013). No such significant association was found for fathers. The children of mothers with tertiary education were 36% less likely (OR = 0.64, *p* < 0.001) to be in the excessive screen time group compared to children of mothers with primary education.

Compared to low-income families, children in high and middle-income families were significantly less likely to not comply with the recommendations for screen time (*p* = 0.003; *p* = 0.014). There was no significant correlation between the physical activity of adults and the screen time of children. Children who adhered to the recommended amount of physical activity were 15% less likely (OR = 0.85, *p* = 0.004) to exceed the recommended amount of screen time compared to those who did not engage in sufficient physical activity. No significant association was found between children’s screen time and parental risk of T2DM (*p* = 0.099).

The children’s gender (*p* = 0.559), age (*p* = 0.884), and BMI Z-score (*p* = 0.294) showed no significant relationship with their time spent in front of a screen. However, children with less than 7 h/week of physical activity had a significantly lower proportion of children who spent more than 2 h in front of a screen (*p* = 0.004).

## 4. Discussion

In our study, we investigated children’s screen time and its association with various factors in six European countries. These factors included parental sociodemographic characteristics, BMI, physical activity, and family risk status for T2DM. Additionally, we examined the association of screen time with children’s gender, physical activity, and BMI.

In the six countries surveyed, 53% of children aged 6–9 spent more than 2 h per day in front of a screen. This figure is only 7% lower than that reported in another large European study for the same age group, the WHO European Childhood Obesity Surveillance Initiative (COSI) 2015–2017 [24]. Significant differences were seen between countries, with notably higher screen use in Belgium, Finland, and Bulgaria compared to Hungary.

Our results indicate that among the sociodemographic factors of parents, both the age and education level of the mother, as well as the father’s education level, were significantly associated with children’s screen time. Specifically, maternal age of 45–54 years and either maternal or paternal tertiary education were linked to reduced odds of children exceeding 2 h of screen time per day, compared to their respective reference groups. These findings align with previous research [16,40,41]. The significant impact of maternal age in the 45–54 years bracket is likely attributable to a higher proportion of mothers with tertiary education in this age group compared to those in lower or higher age groups.

Consistent with previous findings, the socioeconomic status of families significantly influenced children’s screen time [16,40,41]. Middle- and high-income levels were inversely associated with children exceeding 2 h of screen time per day.

Maternal overweight/obesity, which is closely linked to lifestyle habits, increased the likelihood of children having higher than recommended screen time. This association was not seen in fathers. Similarly, parental physical activity did not correlate with excessive screen time in children. Previous studies have also clearly proven that children of mothers with low educational attainment tend to have higher screen time than recommended [42,43,44]. This could be attributed to mothers with lower educational levels spending less time with their children, adopting less healthy lifestyles, and imposing fewer restrictions on their children’s screen time. These factors can negatively impact their children’s screen time [42,44,45,46].

Among the variables examined in the children, physical activity was associated with screen time, with lower physical activity levels correlating with increased odds of favorable screen time. Previous studies suggest that the relationship between screen time and physical activity is not uniformly consistent. For instance, a Spanish study discovered positive relationships between screen time and light physical activity but a negative correlation between moderate-to-vigorous physical activity and screen time among adolescents [47]. Similarly, a Brazilian study indicated that inadequate physical activity increased the likelihood of high screen time among adolescents [48]. Our results might be explained by a previous study’s findings, which suggested that productive sedentary behaviors (such as reading, homework, and computer-related work) were associated with increased physical activity [49]. In contrast, time spent watching TV and playing video games did not correlate with reduced physical activity [49]. Due to this controversial result, which was also found in the previously mentioned studies, children who were more physically active, while more likely to spend time in front of a screen, presumably tended to use this time for more productive activities, such as doing homework or computer practice. 

In our sample, there was no significant association between the children’s gender or BMI and their screen time. Previous studies have also shown inconsistent results mainly regarding children’s gender [16,18,50,51]. For instance, one study analyzing pooled data from several countries found no significant gender-related differences in children’s screen time, and while a positive association with BMI was noted, there was considerable heterogeneity between surveys in both aspects [16]. However, one meta-analysis conclusively found that being overweight/obese significantly increases the likelihood of screen time exceeding 2 h per day, and another one found that eating during TV viewing increases the risk of being overweight/obese [18,51]. 

Our study had several strengths and limitations. One strength of our study is its use of standardized methodology across six European countries and the determination of children’s BMI based on real-world data. The large sample size enhances the reliability of our results. However, a limitation of our survey is its reliance on self-reported questionnaires, which may lead to the potential for recall and social desirability biases. Self-reported weight and height can also lead to potential bias in the BMI classification. Additionally, taking into account that it was a cross-sectional study, it was not possible to find causality.

## 5. Conclusions

Screen overuse among children is also prevalent in the surveyed countries. Our study results highlight that the most influential factors contributing to children’s excessive screen time are the mother’s education level and the family’s income, indicative of the family’s socioeconomic status. Our results also underscore that the family’s socioeconomic status is a more important factor in the determination of screen time than the country’s socioeconomic status. Additionally, maternal BMI and children’s physical activity showed a positive correlation with extended screen time. Further research is needed to investigate why screen time is predominantly influenced by maternal education and family income. Since the parents serve as role models for their young children [52,53], we have to focus on parents’ education, especially mothers from low socioeconomic families, in the interventional programs targeting to mitigate screen time in this group.

## Figures and Tables

**Table 1:** Descriptive statistics by screen time status of the children.

Variable	Level	Screen Time (*n*,%)		*p*-Value
		Ideal (<2 h/day)	No Ideal (≥2 h/day)	
Country	Belgium	572 (43%)	771 (57%)	<0.001
	Finland	556 (43%)	734 (57%)	
	Greece	951 (52%)	872 (48%)	
	Hungary	692 (52%)	642 (48%)	
	Bulgaria	885 (41%)	1298 (59%)	
	Spain	627 (59%)	437 (41%)	
Family at risk of developing type 2 diabetes mellitus *	Non high risk	3358 (48%)	3673 (52%)	0.192
	High risk	925 (46%)	1081 (54%)	
Age of mother	<45 year	3721 (47%)	4198 (53%)	0.052
	45–54 year	418 (52%)	390 (48%)	
	55–64 year	2 (29%)	5 (71%)	
	>64 year	1 (33%)	2 (67%)	
BMI mother **	Not overweight (BMI < 25)	2896 (49%)	3029 (51%)	<0.001
	Overweight or obese (BMI ≥ 25)	1203 (44%)	1507 (56%)	
Education mother	Primary education level (≤9 year)	286 (46%)	342 (54%)	<0.001
	Secondary education level (10–14 year)	1309 (44%)	1690 (56%)	
	Tertiary education level (≥15 year)	2470 (50%)	2484 (50%)	
Age of father	<45 year	2795 (47%)	3129 (53%)	0.439
	45–54 year	746 (49%)	763 (51%)	
	55–64 year	54 (49%)	57 (51%)	
	>64 year	6 (55%)	5 (45%)	
BMI father **	Not overweight (BMI < 25 kg/m^2^)	1225 (49%)	1259 (51%)	0.061
	Overweight or obese (BMI ≥ 25 kg/m^2^)	2328 (47%)	2624 (53%)	
Education father	Primary education level (≤9 year)	313 (47%)	354 (53%)	<0.001
	Secondary education level (10–14 year)	1457 (44%)	1870 (56%)	
	Tertiary education level (≥15 year)	1793 (51%)	1727 (49%)	
Income status	Low	1911 (46%)	2246 (54%)	0.035
	Middle	1194 (47%)	1343 (53%)	
	High-income	982 (49%)	1002 (51%)	
Adult physical activity	Ideal (≥150 min/week)	1931 (47%)	2140 (53%)	0.996
	No ideal (<150 min/week)	2333 (47%)	2586 (53%)	
Child gender	Female	2178 (47%)	2454 (53%)	0.484
	Male	2087 (48%)	2283 (52%)	
Child physical activity	Ideal (≥7 h/week)	2660 (47%)	2955 (53%)	0.881
	No ideal (<7 h/week)	1601 (48%)	1767 (52%)	
Screen time		4283 (47%)	4754 (53%)	

* At least one parent had a Findrisc score of a minimum of 9. ** BMI (body mass index) was calculated according to the WHO (World Health Organization) criteria.

**Table 2:** Age and gender of children.

Variable	*n* *	Mean	S.D. **	0.25	Quantiles Median	0.75
Age years	12,047	8.21	1.00	7.46	8.16	8.93
BMI Z-scores	12,030	0.56	1.09	−0.19	0.47	1.29

* *n*: number of cases; ** S.D.: standard deviation.

**Table 3:** Factors that influenced screen time among the children based on the multivariate logistic regression model.

Factor (Stratum, If Any)	Level	Adjusted Odds Ratio [95% CI]	*p*-Value
Country	Hungary	Ref	
	Belgium	1.86 [1.52–2.29]	<0.001
	Finland	1.76 [1.43–2.16]	<0.001
	Greece	1.02 [0.85–1.22]	0.836
	Bulgaria	1.91 [1.59–2.29]	<0.001
	Spain	1.02 [0.81–1.28]	0.850
Family at risk of developing type 2 diabetes mellitus *	Non high risk	Ref	
	High risk	1.11 [0.98–1.25]	0.099
Age of mother	<45 year	Ref	
	45–54 year	0.81 [0.66–0.98]	0.033
	55–64 year	0.43 [0.03–7.07]	0.558
	≥65 year	-	-
BMI mother **	Not overweight (BMI < 25 kg/m^2^)	Ref	
	Overweight or obese (BMI ≥ 25 kg/m^2^)	1.15 [1.03–1.29]	0.013
Education mother	Primary education level (≤9 year)	Ref	
	Secondary education level (10–14 year)	0.83 [0.65–1.06]	0.137
	Tertiary education level (≥15 year)	0.64 [0.49–0.82]	0.001
Age of father	<45 year	Ref	
	45–54 year	0.99 [0.87–1.14]	0.945
	55–64 year	1.13 [0.74–1.73]	0.580
	≥65 year	0.53 [0.08–3.28]	0.496
BMI father **	Not overweight (BMI < 25 kg/m^2^)	Ref	
	Overweight or obese (BMI ≥ 25 kg/m^2^)	1.05 [0.94–1.17]	0.388
Education father	Primary education level (≤9 year)	Ref	
	Secondary education level (10–14 year)	1.16 [0.94–1.43]	0.172
	Tertiary education level (≥15 year)	0.99 [0.79–1.26]	0.996
Income status	Low	Ref	
	Middle	0.85 [0.75–0.97]	0.014
	High-income	0.80 [0.69–0.93]	0.003
Adult physical activity	Ideal (≥150 min/week)	Ref	
	No ideal (<150 min/week)	0.96 [0.86–1.07]	0.469
Child gender	Female	Ref	
	Male	1.03 [0.93–1.14]	0.559
Child physical activity	Ideal (≥7 h/week)	Ref	
	No ideal (<7 h/week)	0.85 [0.76–0.95]	0.004

* At least one parent had a Findrisc score of a minimum of 9. ** BMI (body mass index) was calculated according to the WHO (World Health Organization) criteria. CI: confidence interval. Ref: reference.

## Data Availability

The data presented in this study are available upon request from the corresponding authors. The data are not publicly available due to restrictions eg privacy or ethical.
